# Distinct Epitopes on CD13 Mediate Opposite Consequences for Cell Adhesion

**DOI:** 10.1155/2018/4093435

**Published:** 2018-03-29

**Authors:** Claudia A. Garay-Canales, Ileana Licona-Limón, Enrique Ortega

**Affiliations:** Departamento de Inmunología, Instituto de Investigaciones Biomédicas, Universidad Nacional Autónoma de México, Apartado Postal 70228, Ciudad Universitaria, 04510 Ciudad de México, Mexico

## Abstract

CD13 is a membrane glycoprotein with aminopeptidase activity, expressed on several cell types, including myeloid cells (dendritic cells, monocytes, macrophages, neutrophils, etc.). CD13 participates in several functions such as proteolytic regulation of bioactive peptides, viral receptor, angiogenesis, and tumor metastasis. CD13 has also been proposed to participate in cell adhesion, as crosslinking of CD13 by certain CD13-specific antibodies induces homotypic aggregation of monocytes and heterotypic adhesion of monocytes to endothelial cells. We generated two monoclonal antibodies (mAbs C and E) that block homotypic aggregation of U-937 monocytic cells induced by CD13-specific mAb 452. Moreover, the mAbs cause detachment of cells whose aggregation was induced by CD13 crosslinking. Both mAbs also inhibit heterotypic adhesion of U-937 monocytes to endothelial cells. mAbs C and E recognize membrane CD13 but bind to epitopes different from that recognized by mAb 452. Crosslinking of CD13 by mAb C or E is required to inhibit adhesion, as monovalent Fab fragments are not sufficient. Thus, C and E antibodies recognize a distinct epitope on CD13, and binding to this epitope interferes with both CD13-mediated cell adhesion and enzymatic activity. These antibodies may represent important tools to study cell-cell interactions mediated by CD13 in physiological and pathological conditions.

## 1. Introduction

Aminopeptidase N (EC 3.4.11.2, APN) is an integral membrane protein with zinc-dependent peptidase activity, first isolated in 1963 by Pfleiderer and Celliers [1, 2]. APN preferentially removes N-terminal neutral amino acids from unsubstituted oligopeptides, amides, or arylamides. Through its peptidase activity, it is known to participate in regulation of the activity of various neuropeptides, as well as vasoactive and chemotactic peptides. APN has been also shown to participate in several other processes, like differentiation, proliferation, apoptosis, motility, chemotaxis, antigen presentation, and tumor cell invasion, among others [3]. Participation of APN in these processes not always depends on its peptidase activity. In 1989, Look et al. established the identity of APN with the myeloid marker CD13 [4].

Structurally, APN/CD13 is a membrane protein of 967 amino acids which has a large extracellular portion containing the enzymatic active site, a transmembrane domain, and a short cytoplasmic tail. Crystallographic structure of the large extracellular portion of CD13/APN reveals that it has a seahorse shape, with four distinct domains: head, side, body, and tail [5, 6]. CD13 is expressed on the cell membrane as a highly glycosylated dimer of two noncovalently associated subunits of 160 kDa. A soluble form of CD13 is also detectable in plasma/serum and urine [7, 8].

In homeostasis, CD13 is expressed in epithelial, endothelial, and fibroblast cell types; within the hematopoietic compartment it is expressed on stem cells and on cells of the granulocytic and monocytic lineages at distinct stages of differentiation and has thus been considered a differentiation marker [9]. Aberrant expression of CD13 is observed in many diseases, and a high expression of CD13 in melanoma, renal, pancreas, colon, prostate, gastric, and thyroid cancer cells has been associated with a poor prognosis [10]. Overexpression of CD13 has been also observed in inflammatory diseases, such as in alveolar macrophages from collagen vascular disease patients with interstitial lung disease [11] and in synovial fibroblasts from rheumatoid arthritis patients [12].

CD13 is considered a moonlighting protein, because it has multiple functions that are apparently not related mechanistically. Along with its enzymatic activity, CD13 also participates in angiogenesis [13, 14], as a receptor for some group 1 coronaviruses [15], and in cholesterol uptake [16]. Also, we have previously reported that CD13 is involved in adhesion of monocytes [17] and that CD13 is a phagocytic receptor [18]. Participation of CD13 in adhesion processes of monocytes was demonstrated by showing that crosslinking of CD13 with a monoclonal antibody (mAb) (clone 452) resulted in the homotypic aggregation (HA) of U-937 human monocytic cells through a signal transduction dependent process, which required metabolic energy [17]. Later, it was shown that CD13 crosslinking by mAb 452 also induces monocyte adhesion to endothelial cells [19]. In the later study, it was suggested that CD13 directly mediates cell-cell interactions, as adhesion can be blocked by soluble CD13, and activated monocytes can adhere to immobilized purified recombinant CD13 [19]. Demonstration of the involvement of CD13 in mediating monocyte adhesion* in vivo* was given by Ghosh et al. [20], who reported that peritoneal monocytes, macrophages, and dendritic cells were significantly decreased in inflammatory exudates from CD13-KO mice compared to wild-type mice. They also showed, using a model of adoptive transfer of myeloid cells from wild-type and CD13-KO mice into either wild-type or CD13-deficient mice, that thioglycollate-induced migration to the peritoneal cavity was significantly reduced in the absence of CD13 expression in either monocytes or endothelial cells. Subramani et al. [21] found that, in U-937 monocytic cells, CD13 clustering induces adhesion through a mechanism that involves activation of focal adhesion kinase (FAK), Src, and ERK protein kinases and phosphorylation of a tyrosine in the short cytoplasmic tail of CD13. Mutation of this tyrosine, or the use of chemical inhibitors of tyrosine kinases, abrogates cell adhesion to endothelial cells* in vitro* and impairs monocyte trafficking to the inflamed peritoneum* in vivo *[21].

Only certain anti-CD13 mAbs are able to induce monocyte adhesion, suggesting that the epitope recognized by each mAb is important in determining its ability to initiate signal transduction events leading to adhesion [17]. We report here the characterization of the adhesion-related properties of two new monoclonal anti-human CD13 antibodies. These mAbs inhibit CD13 enzymatic activity and are able to inhibit HA of monocytes, as well as the heterotypic aggregation of monocytes to endothelial cells, induced by the activating mAb 452. These antibodies may represent important tools to study cell-cell interactions mediated by CD13 in physiological and pathological conditions.

## 2. Materials and Methods

### 2.1. Cells and Antibodies

#### 2.1.1. Cell Lines

The human promonocytic cell lines U-937 and THP-1, the murine myeloma Ag8 cells (obtained from the American Type Culture Collection, ATCC, Manassas, VA), and THP-1 L2 cells (THP-1 cells with low expression of CD13 [18]) were cultured in RPMI-1640 medium (Gibco by Life Technologies, NY, USA). HMEC-1 microvasculature endothelial cells (originally from ATCC, obtained from Dra. Dolores Correa, Instituto Nacional de Pediatría, México) were cultured in MCDB-131 medium supplemented according to the manufacturer's instructions (Invitrogen, Carlsbad, CA, USA). HEK-293 cells (ATCC CRL-1573™) or HEK-ANPEP cells (HEK-293 cells expressing human CD13-GFP, obtained as previously described [18]) were cultured in DMEM-high glucose (Invitrogen, CA, USA). J744-hCD13 mouse macrophages expressing human CD13 [18] were cultured in DMEM-high glucose (Gibco by Life Technologies, USA). All media were supplemented with 10% heat-inactivated FBS (Invitrogen, Carlsbad, CA, USA), 2 mM L-glutamine, 100 *μ*g/ml streptomycin, 100 U/ml penicillin (Sigma-Aldrich, St. Louis MO, USA), 1 mM sodium pyruvate solution, and 1% MEM nonessential amino acids solution (100x) (Gibco by Life Technologies, NY, USA). Cultures were maintained at 37°C in a humidified atmosphere with 6% CO_2_.

#### 2.1.2. Antibodies

Hybridoma cells producing monoclonal antibodies C (mAb C) (IgG1) or E (mAb E) (IgG2b) were obtained by fusion with PEG 1550 (SERVA, Heidelberg, Germany) of Ag8 myeloma cells with splenocytes from a 4-week-old female BALB/c mouse immunized intraperitoneally with U-937 cells aggregated after stimulation with anti-CD13 mAb 452 (1 *µ*g/ml for two hours). Immunizations were given every two weeks during 2 months. Two additional intraperitoneal immunizations with aggregated cells were applied 3 and 4 days before the fusion. Animal handling and sacrifice were conducted under the guidelines established by the Committee on the Care and Use of Experimental Animals of the Instituto de Investigaciones Biomédicas, Universidad Nacional Autónoma de México (UNAM). Hybridomas were selected based on the ability of the secreted mAb to inhibit homotypic aggregation (HA) of U-937 cells induced by mAb 452 [17]. For these assays, U-937 cells (1 × 10^5^ cells in 50 *µ*l RPMI 1640) were placed in flat-bottom wells of a 96-well culture plate, an equal volume of medium or supernatant from the hybridomas was added to each well, and cells were incubated for 1 h at 37°C. Activating mAb 452 was then added (at a final concentration of 1 *μ*g/ml) and cells were incubated for two additional hours. Inhibition of aggregation by supernatants was detected visually in an Axiovert 25 inverted microscope (Zeiss, Germany). Hybridomas secreting antibodies able to inhibit HA were selected for cloning by limiting dilution, expanded, and characterized. Isotype of the monoclonal antibodies was determined using the mouse immunoglobulin isotyping kit (Invitrogen, CA, USA). MAb C is an IgG1 and mAb E an IgG2b. Monoclonal antibodies were purified by affinity chromatography on protein G- or protein A-agarose columns.

Murine monoclonal anti-human CD13 (clone 452, IgG1) was purified from culture supernatants of the hybridoma, kindly donated by Dr. Meenhard Herlyn (The Wistar Institute of Anatomy and Biology, Philadelphia, PA). Fab fragments of antibodies were prepared with immobilized Ficin (IgG1) or papain (IgG2b) (Pierce, Rockford, IL). APC-labeled mAb anti-human CD13 (Clone WM-15) was obtained from Biolegend (USA).

F(ab)′_2_ fragments of goat anti-mouse IgG were purchased from Jackson ImmunoResearch (West Grove, PA, USA). Goat anti-mouse-FITC, used as secondary antibody for immunostaining, was from Zymed (ThermoFisher, Rockford, IL, USA). Purified anti-CD13 mAbs C, E, or 452 were directly labeled with FITC (Sigma, St Louis, MO, USA) in our laboratory.

### 2.2. Inhibition of Homotypic Aggregation and Detachment Assays

Monocytic U-937 cells (1 × 10^5^ in 50 *µ*l RPMI 1640) were placed in flat-bottom wells of 96-well culture plates (Corning, NY, USA) and were incubated with the indicated concentrations of mAbs for 60 min at 37°C in a humidified atmosphere with 6% CO_2_. Activating anti-CD13 mAb 452 (1 *µ*g/ml) was added to each well, and cells were incubated at 37°C for two additional hours. Cell aggregation was evaluated by light microscopy. Images of each well were captured with a digital camera attached to a Zeiss Axiovert 25 inverted microscope, and cells in aggregates were quantified using the colony-counting function of Quantity One software (Bio-Rad, Hercules, CA). Data are presented as aggregation indexes (AI) = [number of cells detected by software as blue colonies in each image (aggregated cells)/total number of cells detected × 100], as previously described [17]. The effect of inhibitor antibodies is expressed as percentage of control AI. We adjusted the software to detect as blue colonies all cells in aggregates formed by more than three to four cells with optical densities (OD) high enough to avoid erroneous detection of two close cells as aggregates. AI obtained by quantification with the software is lower than, but comparable with, percentage of aggregation obtained by visual counting.

To evaluate the ability of mAbs C and E to produce detachment of homotypic aggregates, U-937 cells were incubated with HA-inducing mAb 452 for 2 hours, to induce aggregation. The mAbs C (0.3 *μ*g/ml), E (0.1 *μ*g/ml), or control IgG (0.3 *μ*g/ml) were added, and AIs at different times were determined as described above.

### 2.3. Immunoprecipitation and Immunoblotting

To identify the protein(s) recognized by HA-inhibiting mAbs, U-937 cells were lysed in 1% Nonidet P-40 (NP-40) lysis buffer with protease and phosphatase inhibitors. Lysates were incubated 15 min on ice and were cleared by centrifugation at 14,000 rpm for 15 min at 4°C. Supernatants were incubated for 3 hours with protein A/G agarose beads coated with mAbs C, E, and 452 or IgG control. After washing, immunoprecipitates or total cell lysates were boiled in reducing Laemmli sample buffer, separated by SDS-PAGE (7.5% acrylamide) and electroblotted onto nitrocellulose membranes, followed by probing with the relevant primary antibody C, E, or 452 (2 *µ*g/ml) and the HRP-conjugated secondary antibody (goat anti-mouse IgG-HRP, Zymed-ThermoFisher, Rockford, IL, USA) diluted 1 : 5000 and detected using SuperSignal West Pico Chemiluminescent Substrate (Thermo Scientific, Rockford IL, USA). Human monocytes isolated from healthy donors and HMEC-1 endothelial cells were similarly lysed, immunoprecipitated with mAb C, resolved in SDS-PAGE, electroblotted onto nitrocellulose membranes, and probed with mAb 452 and HRP-conjugated secondary antibody, as described above.

### 2.4. Mass Spectrometry and Identification by MALDI TOF/TOF

After separation by SDS-PAGE electrophoresis, sections of gel containing the bands of interest were excised, washed, and in-gel digested for 6 hrs at 37°C using modified porcine trypsin (Promega, Madison, WI). The resulting peptides were extracted in two steps, partially dried, and dissolved in 0.1% TFA (Aldrich, Milwaukee, WI). Salts were removed using C18-ZipTip® (Millipore, Bedford, MA) solid phase extractions into 10 *µ*L of 1 : 1 TFA : acetonitrile, and peptides were analyzed by MALDI TOF/TOF spectrometry in the laboratory of Dr. Jose Luis Gallegos at INMEGEN, México. Database searching and protein identification were performed with the MALDI TOF/TOF spectra datasets using the MASCOT search algorithm against the taxonomy of* Homo sapiens* (human) in the nonredundant NCBI (NCBInr) database (version 1.6b9; MatrixScience, London, UK, available at http://www.matrixscience.com/).

### 2.5. Binding and Internalization Assays

Binding of anti-CD13 mAbs to different cells was evaluated by flow cytometry. Cells were incubated with the indicated concentration of anti-CD13 antibody for 30 min at 4°C. The cells were washed twice and secondary antibody (goat anti-mouse Ig-FITC) was added for 30 min at 4°C. After washing, cells were fixed with 1% PFA in PBS for 20 min and analyzed in an Applied Biosystems® Attune® Acoustic Focusing Cytometer (Carlsbad, CA USA). Internalization of CD13 induced by anti-CD13 mAbs was evaluated by incubating the cells with the indicated concentrations of anti-CD13 mAb for 2 h at 37°C or 4°C. Cells were washed and fixed with 1% PFA in PBS for 20 min. After washing, binding of a noncompeting anti-CD13 mAb labeled with FITC was evaluated by flow cytometry.

### 2.6. Determination of Aminopeptidase N Enzymatic Activity

APN enzymatic activity was determined by colorimetric measurement of the hydrolysis of the substrate L-alanine 4-nitroanilide hydrochloride (Sigma, USA), as described previously [17]. Briefly, 1 × 10^5^ cells (in 50 *µ*l PBS with 10% FBS) were placed in flat-bottom wells of 96-well culture plates and were preincubated with the indicated mAb or bestatin for 1 h at 37°C. Bestatin (Santa Cruz Biotechnology, Dallas TX, USA), a well-known CD13 inhibitor, was added at the maximal inhibitory doses of 4 *µ*g/ml for U-937 cells and 40 *µ*g/ml for HEK-ANPEP. After incubation of the cells with the mAbs or with bestatin, substrate was added to a final concentration of 6 mM and the plates were incubated for 1 h at 37°C. Cells were pelleted, and the absorbance of the supernatants at 405 nm was determined immediately. Determinations were performed in triplicate wells. Data are presented as percentage of the activity of control cells, which were not incubated with mAbs or bestatin before addition of the substrate.

### 2.7. Competitive Binding Assays

U-937 cells (2 × 10^5^) were incubated in FACS buffer (PBS with 5% FBS, 0.1% sodium azide) with saturating concentrations of the indicated competing unlabeled anti-CD13 antibody, for 30 min at 4°C. Cells were washed and saturating amounts of the fluorescently labeled antibody were added and incubation continued for another 30 min at 4°C. Cells were fixed in 1% paraformaldehyde and analyzed by flow cytometry (Applied Biosystems Attune Acoustic Focusing Cytometer, Carlsbad, CA USA).

### 2.8. Tyrosine Phosphorylation of CD13

U-937 cells were incubated in serum-free RPMI-1640 medium for 2 hours at 37°C. Cells were adjusted to 2 × 10^7^ per sample and were treated with activating or inhibitory anti-CD13 monoclonal antibodies 452 or mAb C or E for the indicated time intervals at 37°C, in culture medium with 3.5% FBS. After stimulation, the reaction was stopped by adding an equal volume of ice-cold TBS, and cells were washed once. Monocytes were lysed with 1% lysis buffer (Tris-HCl 20 mM, NaCl 150 mM, pH = 7.4 and 1% NP-40) with protease and phosphatase inhibitors for 15 min at 4°C. Lysates were cleared by centrifugation at 20,817*g* for 15 min. Lysates were precleared with Sepharose for 60 minutes at 4°C, followed by overnight incubation at 4°C with protein G agarose beads that were previously incubated with a subsaturating concentration of mAb C (20 *µ*g/20 *µ*l beads). After washing, beads were resuspended in Laemmli sample buffer and incubated for 5 min in a boiling water bath. Samples were resolved in 7.5% SDS-PAGE, electrotransferred onto nitrocellulose membranes, and blocked overnight at 4°C (3% BSA in TBS-tween 0.1%, pH = 7.4).

Phosphorylation was detected in blots probed with a mixture of anti-phosphotyrosine murine antibodies (PY-20 Santa Cruz, 4G10 Upstate Biotechnology, and AFT-8 kindly donated by Dr. Carlos Rosales, IIBiomédicas, UNAM). To detect CD13, blots were probed with mAb 452 (2 *µ*g/ml). After incubation with the relevant primary antibody and HRP-conjugated secondary goat anti-mouse Ig antibody (1 : 10,000), proteins were visualized using SuperSignal West Pico Chemiluminescent Substrate (Thermo Scientific). Blots were digitalized using Gel Doc 2000 by Bio-Rad (Bio-Rad, Hercules, CA).

### 2.9. Determination of Intracellular [Ca^2+^] by Flow Cytometry

Monocytic U-937 cells were washed once with RPMI-1640 with 2% FCS. The cells were then resuspended in RPMI-1640 (5 × 10^6^ cells/ml) and incubated with 2.5 *µ*M Fluo-3-AM and 4.6 *µ*M Fura Red (Invitrogen, Molecular Probes), with gentle constant stirring for 45 min at 37°C in darkness. Cells were washed twice with RPMI without serum. Cells were then incubated for additional 45 minutes in RPMI without serum, at room temperature with gentle constant stirring in darkness. After washing, the cells were resuspended in RPMI without serum and maintained at 4°C until measurements. Flow cytometric analysis of intracellular [Ca^2+^] was carried out in a FACScalibur flow cytometer (Becton–Dickinson, Heidelberg, Germany) with a laser excitation wavelength of 488 nm. Fluorescence emissions were collected through band-pass filters (FL1 for Fluo-3, 530/30 nm; FL3 for Fura Red, 670 LP). At the indicated times after obtaining a stable baseline, anti-CD13 mAbs C, E, or 452, anti-Fc*γ*RI mAb 32.2, or a control IgG were added at final concentrations of 2 *µ*g/ml. After data collection for 3 min, F(ab)′_2_ fragments of goat anti-mouse Ig were added at a final concentration of 12 *µ*g/ml. These concentrations of primary and secondary antibodies produced the maximal amplitudes of calcium signals. Fluorescence intensity data are depicted as the ratio of the fluorescence intensities (FL1/FL3) versus time. Maximum fluorescence was obtained after addition of Ionomycin (100 nM) as control.

### 2.10. Cell Adhesion Assays

Adhesion of HEK-ANPEP-GFP to fibronectin-coated wells was evaluated as described previously [19]. In brief, HEK-ANPEP-GFP cells were treated or not with different doses of anti-CD13 mAbs 452, WM-15, C, or E, for 30 min at 37°C. Cells were washed and allowed to adhere to fibronectin-sensitized (Calbiochem, San Diego CA, USA) and BSA-blocked microwell plates for 15 min at 37°C. After gentle washing, fluorescence was determined at 485/530 nm in a Cytation 3 microplate spectrofluorometer (Bio-Tek, Winooski, VT, USA).

For heterotypic adhesion assays, human microvasculature endothelial cells HMEC-1 (kindly obtained from Dra. Dolores Correa, Instituto Nacional de Pediatría, Mexico) were cultured in flat-bottom 48-well plates (25,000 cells/well) for 48 hrs at 37°C. CFSE-labeled U-937 monocytic cells were treated or not with anti-CD13 mAbs 452, C, or E (10 *µ*g/ml) for 3 h at 37°C, washed, and added to the wells containing the monolayers of HMEC-1 cells. After incubation for 15 min at 37°C, plates were gently washed with warm RPMI without phenol red and 50 *µ*l of the same medium was added to each well. Fluorescence (from the CFSE-labeled U-937 cells that remained adhered to HMEC-1 cells) was determined at 485/530 nm in a Modulus™ II Microplate Multimode Reader (Turner BioSystems, Inc, USA).

## 3. Results

We have previously reported that crosslinking of CD13 by certain CD13-specific mAbs induces homotypic aggregation (HA) of U-937 human monocytic cells [17]. The HA is an active phenomenon and not a simple aggregation induced by binding of bivalent mAb 452 molecules to CD13 on different cells, as it does not take place at 4°C, and is inhibited by pharmacological inhibitors of tyrosine- and mitogen-activated protein kinases and by the glycolysis inhibitor 2-deoxyglucose. This phenomenon seems to depend on the epitope recognized by the specific mAb and reflects a change in the adhesive properties of the cell. To study the key players of this process, we generated monoclonal antibodies able to inhibit HA of U-937 cells.

**Figure 1:**
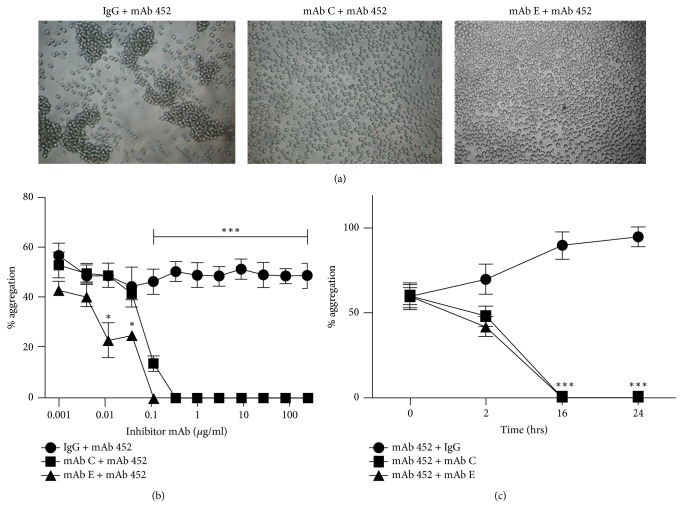
*MAbs C and E inhibit homotypic aggregation of monocytic cells induced by anti-CD13 mAb 452*. (a) U-937 cells in 96-well plates were incubated for 1 h at 37°C with a control IgG, or the monoclonal antibodies C or E, before addition of HA-inducing activating anti-CD13 mAb 452 (1 *µ*g/ml). Representative images of HA were obtained two hours after addition of mAb 452. (b) U-937 cells in 96-well plates were incubated with indicated concentrations of a control IgG, mAb C, or mAb E, for 1 h at 37°, before addition of HA-inducing mAb 452 (1 *µ*g/ml). Percent of aggregated cells was determined 2 h after addition of mAb 452. Percent aggregation (aggregation index, AI) = [number of cells detected by the software as blue colonies in each image (aggregated cells)/total number of cells detected × 100]. (c) Detachment of homotypic aggregates of U-937 cells. U-937 cells were incubated for 2 hrs at 37°C with anti-CD13 mAb 452 to induce aggregation. Then, inhibitory mAbs C or E or control IgG were added (time = 0), and incubation was continued for 24 h at 37°C. Percent aggregation was determined after 2, 16, and 24 h, as described above. The graphs represent the arithmetic mean ± SD of five (b) or three (c) independent experiments. ^*∗*^*p* < 0.05; ^*∗∗∗*^*p* < 0.0001.

We immunized BALB/c mice with U-937 cells previously treated with the HA-inducing anti-CD13 mAb 452. Spleen cells from these mice were fused with Ag-8 myeloma cells, and the resulting hybridomas were selected on the basis of the ability of the secreted mAbs to inhibit HA induced by mAb 452. For the screening, the supernatants of the hybridomas were incubated with U-937 cells in 96-well plates (10^5^ cells/well), for 60 min before addition of the HA-inducing mAb 452. After 2 h of incubation at 37°C, the degree of aggregation in each individual well was assessed under the microscope. We selected two hybridomas, which produce the antibodies named mAb C (IgG1) and mAb E (IgG2b) that are able to inhibit HA induced by mAb 452 (Figure 1(a)), for characterization of the effects of the monoclonal antibodies produced.

### 3.1. mAb C and mAb E Inhibit HA of U-937 Cells in a Time- and Dose-Dependent Manner

To determine the dose-response for inhibition of HA, we incubated U-937 cells in 96-well plates (10^5^ cells/well) with increasing concentrations of purified mAbs C and E or a control mouse IgG1, for 1 h at 37°C before adding an optimal concentration of HA-inducing mAb 452. Percentage of aggregation was determined after 2 h of incubation at 37°C. As shown in Figure 1(b), the inhibition of HA by mAbs C and E was dose-dependent. mAb E was slightly more efficient, being able to completely inhibit HA at a concentration of 0.1 *µ*g/ml, compared to 0.3 *µ*g/ml for mAb C (Figure 1(b)).

Then, we evaluated whether mAbs C and E would have an effect once the homotypic aggregation of U-937 cells induced by mAb 452 was initiated. Thus, we incubated U-937 cells with HA-inducing mAb 452 for two hours, to induce HA. Then, we added mAb C or mAb E (or a control IgG) at final concentrations at which we observed total inhibition of HA, and incubation was continued. We evaluated the degree of cell aggregation at 2, 16, and 24 h after addition of mAbs C and E or the control IgG. As shown in Figure 1(c), at the time that mAbs C or E were added, around 60% of cells were already in aggregates. This fraction of aggregated cells increased over time in the presence of a nonspecific IgG, reaching almost 100% of cells in aggregates at 24 hours. However, in the presence of mAb C or mAb E, not only did the percentage of aggregated cells not increase further, but in fact it decreased. The decrease was already evident after 2 h, and at 16 h of incubation complete separation of the cells was observed, and the cells remained nonaggregated at the 24 h time point (Figure 1(c)). These results suggest that these antibodies are able both to prevent and to revert HA induced by mAb 452.

### 3.2. Inhibitory mAbs C and E Recognize CD13 in Monocytes and Endothelial Cells

**Figure 2:**
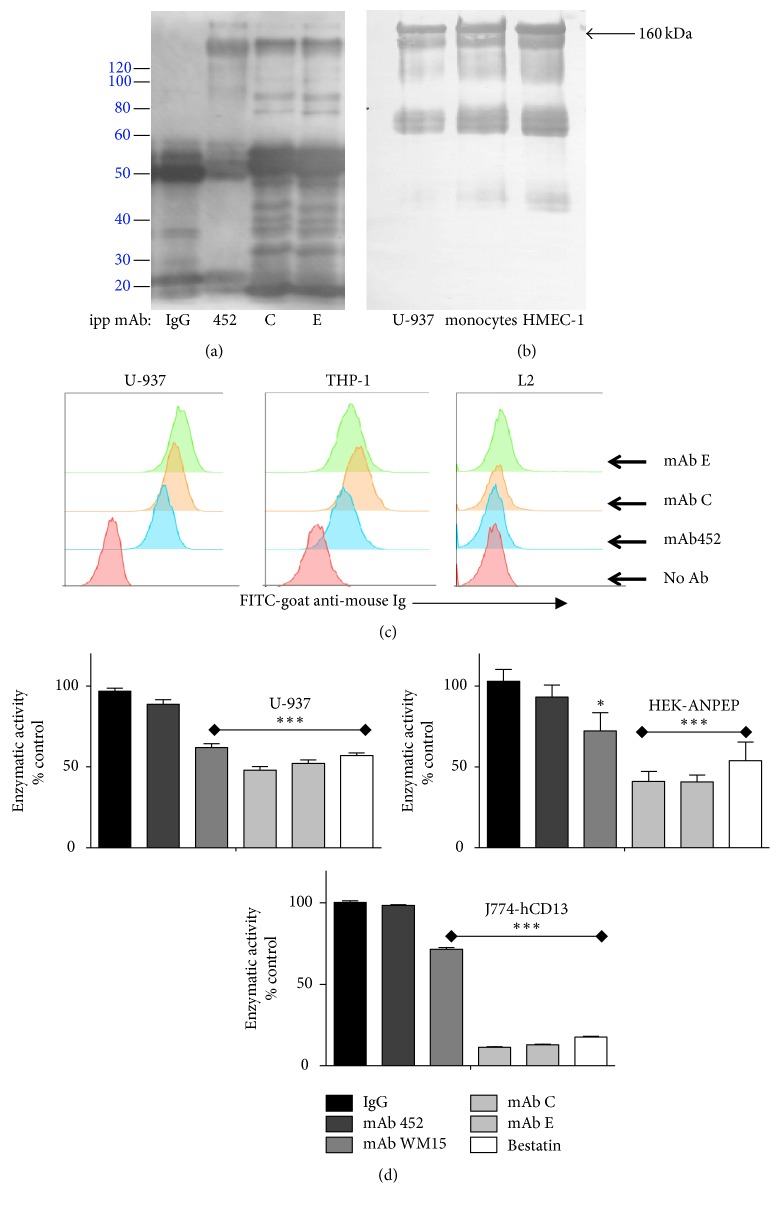
*Inhibitory mAbs C and E recognize CD13 in monocytes and endothelial cells*. (a) Lysates of U-937 cells were immunoprecipitated with mAbs 452, C, E or a control IgG, bound to protein A/G agarose beads. Immunoprecipitates were eluted from beads and resolved in 10% SDS-PAGE, electrotransferred to a nitrocellulose membrane, and probed with mAb C (2 *µ*g/ml) and HRP-conjugated secondary antibody. (b) U-937 cells, human peripheral blood monocytes, or endothelial HMEC-1 cells were lysed, immunoprecipitated with mAb C, electrotransferred to nitrocellulose membranes, and probed with mAb 452 and HRP-conjugated secondary antibody, as described in Materials and Methods. (c) Deficient binding of anti-CD13 antibodies to THP-1 cells transfected with siRNA for CD13 (L2 cells). U937 (left panel), THP-1 (center panel), or L2 cells (right panel) were incubated with the indicated mAb, washed, and incubated with a secondary FITC-labeled goat anti-mouse Ig antibody. (d) Inhibition of Aminopeptidase N enzymatic activity by anti-CD13 mAbs 452, WM-15, C, and E and a control IgG, and by the chemical inhibitor bestatin, in cell lines expressing human APN: U-937 cells, HEK cells transfected with human APN/CD13, and J774 murine cells expressing human APN/CD13. The graphs represent the arithmetic mean ± SD of three independent experiments in each cell line. ^*∗*^*p* < 0.05; ^*∗∗∗*^*p* < 0.0001.

To identify the antigen recognized by the inhibitory mAbs C and E, we conducted immunoprecipitation experiments. Lysates of U-937 cells were immunoprecipitated with mAbs C, E, and 452, or a control IgG. Immunoprecipitates were resolved in SDS-PAGE and electrotransferred to nitrocellulose membranes. Each blot was probed with the corresponding mAb and a secondary antibody. The most prominent of the specific bands in the immunoprecipitates of mAbs C, E, and 452 was a band of 160 kDa. These 160 kDa bands immunoprecipitated by each of the three mAbs (C, E, and 452) were recognized by mAb 452 and also by mAbs C and E (Figure 2(a) shows recognition by mAb C of 160 kDa bands immunoprecipitated by the each of three mAbs). This suggested that mAbs C and E recognize the same protein as mAb 452, that is, APN/CD13. To corroborate the identity of the 160 kDa band precipitated by the three mAbs, we extracted the 160 kDa protein immunoprecipitated by mAbs C, E, or 452, directly from slices of the polyacrylamide gel. The extracted proteins were digested with trypsin and analyzed by MALDI TOF/TOF spectrometry. The results were analyzed with the MASCOT software. This analysis identified APN/CD13 as the immunoprecipitated protein, with scores of 175 for the band precipitated by mAb 452, 274 for the band precipitated by mAb C, and 504 for the one precipitated by mAb E. The minimal score for a positive identification using this strategy is a score of 35.

We evaluated whether mAbs C and E would recognize CD13 on monocytes isolated from healthy donors and on human endothelial cells of the cell line HMEC-1. MAb C (Figure 2(b)) and mAb E (not shown) immunoprecipitated similar bands of about 160 kDa from U-937, human monocytes, and HMEC-1 cells, and these bands are recognized by mAb 452. Together, these data support the conclusion that mAbs C and E recognize APN/CD13.

Further data that support that mAbs C and E recognize APN/CD13 was obtained from experiments comparing binding of mAbs C and E with the binding of mAb 452 to the human monocytic cell lines U-937, THP-1, and a subline of THP-1 cell line with reduced expression of CD13 (cells L2, [18]). As shown in Figure 2(c), mAbs C and E showed binding patterns that are similar to mAb 452: they bind efficiently to U-937 and the parental THP-1 cells, and the three antibodies showed decreased binding to the THP-1 cells modified to express low CD13.

We also evaluated the ability of mAbs C and E to inhibit the enzymatic activity of APN on different cell lines and compared it to the effect of the APN/CD13-specific mAbs 452 and WM-15. As a control, we included bestatin, an inhibitor of APN enzymatic activity. As previously reported, mAb WM-15 significantly inhibited APN enzymatic activity, while mAb 452 had a very small (<10%) effect on the enzymatic activity [17, 22]. mAbs C and E significantly inhibited APN activity in U-937 cells, HEK-293 cells transfected with ANPEP-GFP (HEK-ANPEP), and J774 mouse macrophages transfected with human ANPEP-RFP (J774-hCD13) (Figure 2(d)). In the three cell lines, inhibition of APN enzymatic activity by mAbs C and E was more efficient than inhibition by mAb WM-15, and comparable to the inhibition by bestatin.

### 3.3. MAb C and mAb E Bind to an Epitope on CD13 Different from That Recognized by Activating mAb 452, and Inhibition of HA Is Not due to Changes in CD13 Membrane Expression

**Figure 3:**
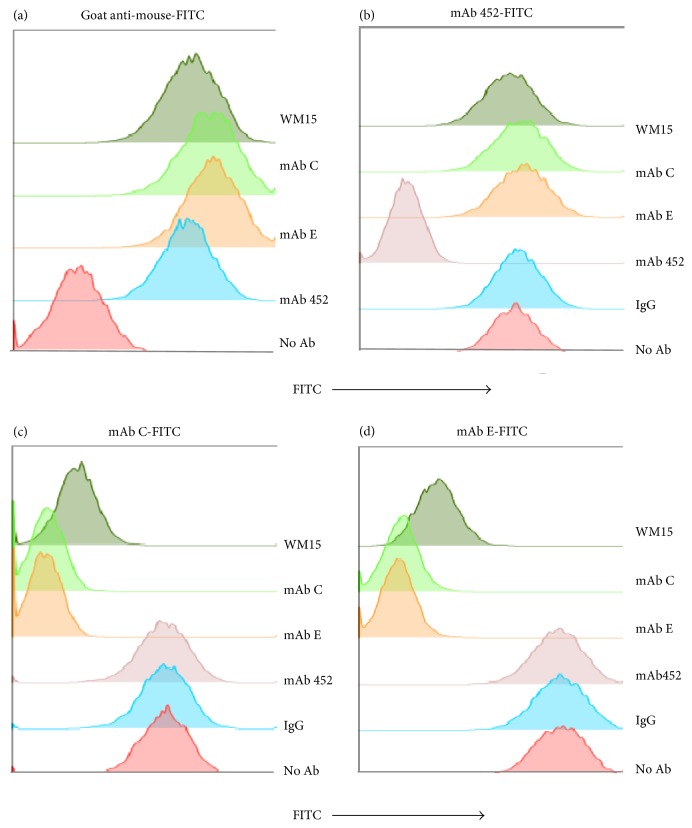
*Inhibitory mAbs C and E recognize an epitope on CD13 that is different from the epitope recognized by mAb 452*. U-937 were incubated with saturating concentrations of unlabeled mAbs WM-15, C, E, and 452, a control IgG, or no antibody, at 4°C. After washing, cells were incubated with (a) goat anti-mouse Ig-FITC, (b) mAb 452–FITC, (c) mAb C-FITC, and (d) mAb E-FITC. Binding of the FITC-labeled antibody was determined by flow cytometry. Representative histograms of 6 different experiments.

The finding that, in contrast to mAb 452 which has a very small effect on APN enzymatic activity, both mAbs C and E inhibited the enzymatic activity considerably might suggest that mAbs C and E bind to a different epitope than 452. In order to determine if the different mAbs bind to different epitopes, we performed competition experiments. First, we verified that purified unlabeled mAbs C and E bind to U-937 cells at similar levels as mAbs 452 and WM15 (Figure 3(a)). Then, we tested the ability of unlabeled mAbs WM-15, C, E, and 452 or a control IgG to inhibit the binding of FITC-labeled mAbs 452, C, and E (Figures 3(b), 3(c), and 3(d)). As expected, binding of mAb 452-FITC was inhibited by mAb 452. However, none of the mAbs WM-15, C, or E, or a control IgG, modified the binding of mAb 452 to the cells (Figure 3(b)). Correspondingly, mAb 452 did not affect binding of FITC-labeled mAb C (Figure 3(c)) or FITC-labeled mAb E (Figure 3(d)) to the cells. These results demonstrate that mAb C and mAb E bind to epitopes on CD13 that are different from the epitope recognized by mAb 452. Interestingly, mAb C and mAb E inhibit each other's binding as efficiently as the corresponding unlabeled mAb, suggesting that these two antibodies bind to the same or to very closely located epitopes on CD13.

**Figure 4:**
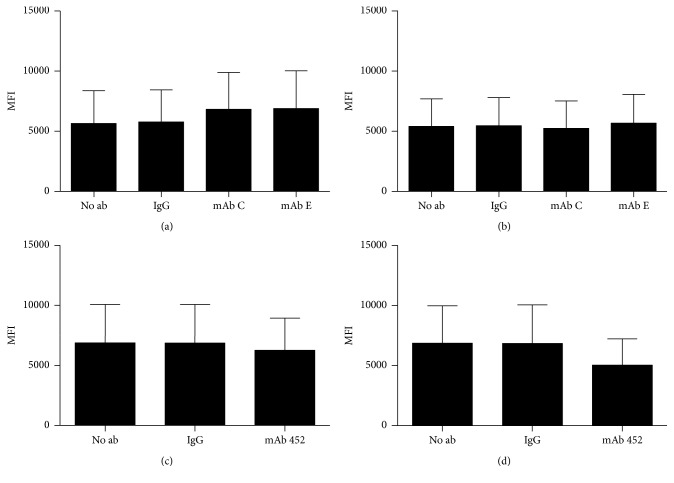
*Binding of mAbs C and E to CD13 do not induce changes in membrane expression of CD13*. (a, b) Human U-937 monocytic cells were incubated for 3 h at 4°C (a) or at 37°C (b), with mAbs C and E or a control IgG, at concentrations at which C and E cause total inhibition of HA. Later, cells were transferred to 4°C and fixed. After fixation, CD13 expression was assessed by the binding of mAb 452-FITC, evaluated by flow cytometry. Data is presented as the average Mean Fluorescence Intensity + SD (*n* = 3). (c, d) U-937 cells were incubated for 3 h at 4°C (c) or at 37°C (d), with mAb 452 (at the optimal concentration for inducing HA), or a control IgG. Later, cells were transferred to 4°C and fixed. After fixation, CD13 expression was assessed by the binding of mAb C-FITC, which was evaluated by flow cytometry. Data is presented as the average Mean Fluorescence Intensity ± SD (*n* = 3). No significant differences were found by ANOVA.

To determine if binding of the mAbs C or E would produce changes in surface expression of CD13, which could be related to their inhibition of HA induced by mAb 452, we incubated U-937 cells with the inhibitory mAbs at 4 or at 37°C, immediately fixed the cells and evaluated binding of anti-CD13 mAb 452-FITC. As shown in Figures 4(a) and 4(b) (and supplementary Figure 1), binding of mAbs C or E to CD13 did not induce changes in CD13 expression on the cell membrane. We conducted the opposite experiments, incubating the cells with mAb 452 at 4 or at 37°C, fixing immediately, and evaluating binding of anti-CD13 mAb C-FITC. When cells were incubated with mAb 452 at 37°C, we found a decrease of 25% in the binding of mAb C-FITC (Figure 4(d) and supplementary Figure 1) or E-FITC (not shown). This decrease, however, is not statistically significant. Since the decrease is not evident when incubation with mAb 452 is carried out at 4°C (Figure 4(c)), it is possible that it reflects a certain amount of CD13 internalization induced by CD13 crosslinking by mAb 452. However, further experiments are required to clarify this issue.

### 3.4. Crosslinking of CD13 by mAbs C and E Is Required to Inhibit HA of Monocytes

**Figure 5:**
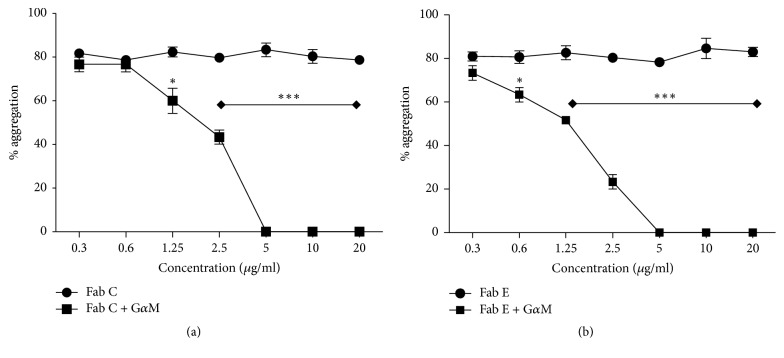
*Inhibition of homotypic aggregation by mAbs C and E requires crosslinking of CD13*. U-937 cells in 96-well plates were incubated with the indicated doses of Fab fragments of inhibitory mAbs C (a) or E (b). Cells were washed, resuspended, and divided into two, a half was incubated with F(ab′)_2_ fragments of goat anti-mouse Ig (G*α*M) and the other half in medium alone. After incubation for 1 h at 37°C, HA-inducing anti-CD13 mAb 452 was added and cells were incubated for 2 additional hours. Percentage of aggregation was determined under light microscopy as described in Materials and Methods. The graphs represent the arithmetic mean ± SD of three independent experiments. ^*∗*^*p* < 0.05, ^*∗∗∗*^*p* < 0.0001.

It has been previously reported that the HA induced by mAb 452 requires the crosslinking of CD13 [17]. Therefore, we sought to determine if binding of mAb C or mAb E to their specific epitope on CD13 is sufficient for inhibition of HA, or if crosslinking of CD13 by these mAbs is required. We performed experiments to evaluate the inhibition of HA by Fab fragments of the inhibitory mAbs, alone or after crosslinking with F(ab)′_2_ fragments of goat anti-mouse Ig (Figure 5). The results indicated that incubation of U-937 monocytes with Fab fragments of mAbs C and E clustered with F(ab)′_2_ fragments of goat anti-mouse Ig is able to inhibit HA as efficiently as the intact mAbs (Figure 1), but monovalent Fab fragments do not inhibit HA at any of the concentrations tested (Figure 5).

### 3.5. Crosslinking of CD13 by mAbs C, E, and 452 Induces a Rise in Intracellular Ca^2+^ and Tyrosine Phosphorylation of CD13

**Figure 6:**
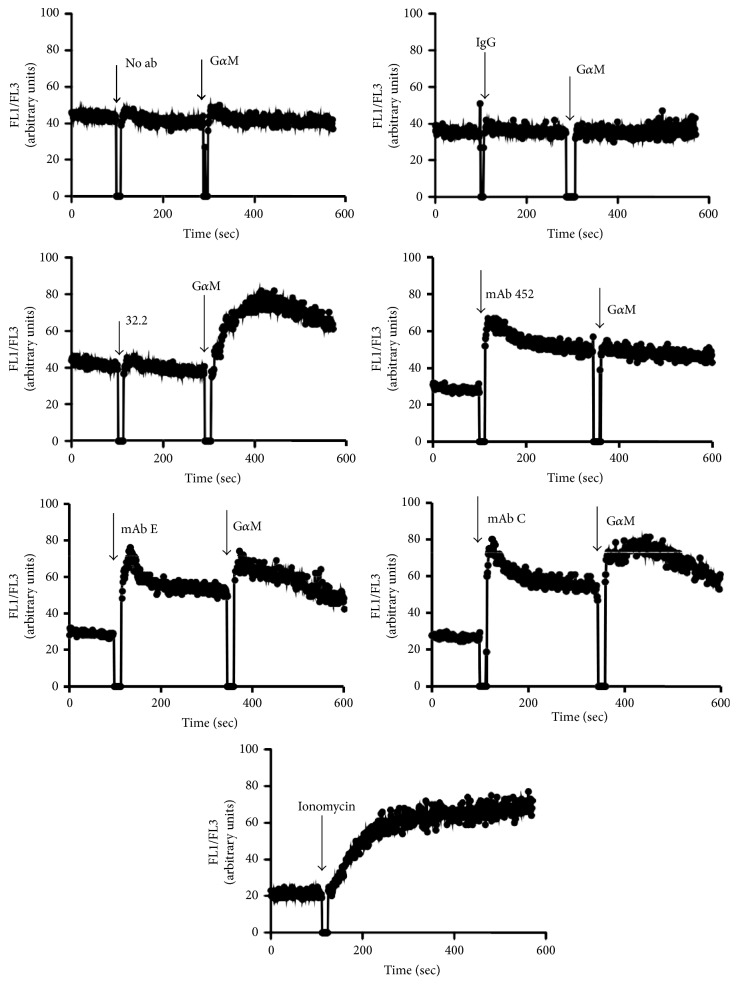
*Changes in intracellular Ca* ^*2*+^ *ions induced by CD13-specific mAbs*. U-937 cells were loaded with Fluo-3 AM and Fura Red. After washing, fluorescence of both indicators was continuously monitored by flow cytometry at 37°C. At the indicated times, cells were stimulated with the indicated antibodies (32.2, 452, C, or E) or a control IgG, and with F(ab′)_2_ fragments of goat anti-mouse Ig (G*α*M). As control, cells were stimulated with secondary antibody alone. Traces shown are representative of 3 independent experiments.

CD13 crosslinking by specific monoclonal antibodies is able to induce signal transduction events, including a rise in intracellular Ca^2+^ ions and tyrosine phosphorylation of several proteins [21, 23]. In order to compare the ability to induce signal transduction of HA-inhibitory mAbs C and E with that of HA-inducing mAb 452, we evaluated changes in intracellular Ca^2+^ ions induced by each of the mAbs. We loaded U-937 cells with the Ca^2+^-sensitive dyes Fluo-3 and Fura Red and monitored changes in intracellular Ca^2+^ after stimulation with intact anti-CD13 mAb 452, mAb C, or mAb E (2 *µ*g/ml) followed by crosslinking of the bound mAbs by addition of F(ab)′_2_ fragments of goat anti-mouse Ig (12 *µ*g/ml), to induce further crosslinking of CD13 (Figure 6). Bivalent mAb 452 induces a very rapid increase in intracellular Ca^2+^ (reaching a maximum in less than 15 sec followed by a gradual decrease) even without further crosslinking by a secondary goat anti-mouse Ig. Addition of a secondary antibody does not affect the gradual decline of intracellular Ca^2+^. Both mAb C and mAb E are also able to induce a fast rise in the intracellular concentration of Ca^2+^, which after an initial peak declines to a plateau higher than the basal levels. Addition of goat anti-mouse Ig secondary antibodies induced a further increase in the concentration of Ca^2+^ ions. As positive control we determined the effect of the anti-Fc*γ*RI mAb 32.2, which by itself was not able to induce a significant increase in intracellular Ca^2+^, but efficiently induced an increase after crosslinking with secondary F(ab)′_2_ fragments of goat anti-mouse Ig, as has been previously reported [24]. Treatment of U-937 with secondary antibody only, or with a control IgG and secondary antibody, did not result in a calcium signal.

**Figure 7:**
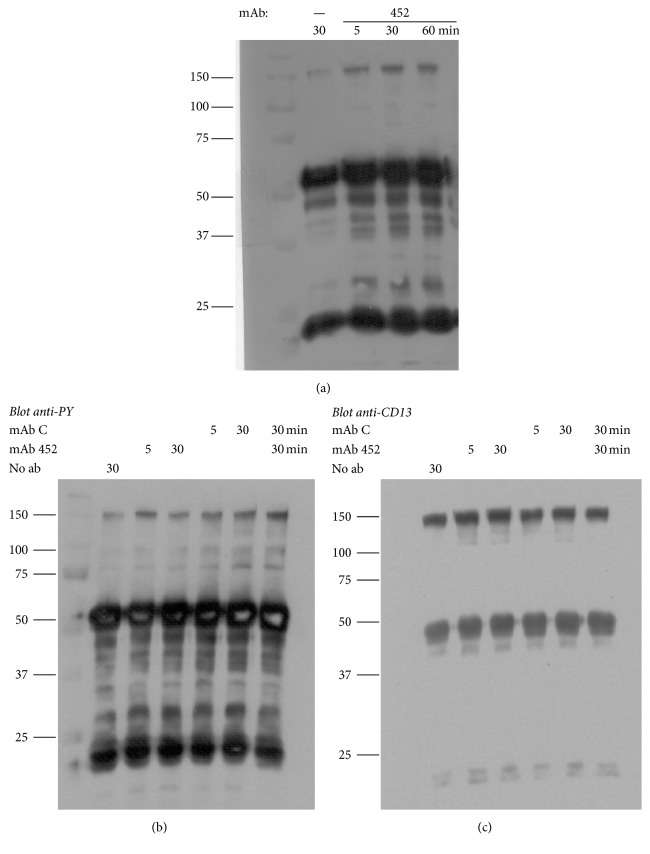
*Effect of anti-CD13 mAbs on CD13 phosphorylation*. (a) Time course of CD13 phosphorylation in U-937 cells stimulated with mAb 452 (1 microgram/ml). After stimulation for 5, 30, and 60 min, cells were lysed and immunoprecipitated with mAb C-protein G agarose. Tyrosine phosphorylation was determined as described in Materials and Methods. (b, c) CD13 phosphorylation induced by anti-CD13 mAbs 452 and C. U-937 cells were stimulated with mAb 452 or mAb C for 5 or 30 min, or with mAb C for 30 min followed by mAb 452 for 30 additional min (last lane). Phosphotyrosine (b) and CD13 (c) were detected as described in Materials and Methods.

Since CD13 crosslinking by mAb 452 has also been shown to induce tyrosine phosphorylation of CD13 [21], we also compared the ability of the anti-CD13 mAbs to promote phosphorylation of CD13. U-937 cells were incubated with intact mAbs 452, C, or E, for 5 and 30 minutes at 37°C, cells were washed with ice-cold buffer and lysed, and CD13 was immunoprecipitated with agarose-protein G beads that have been incubated with subsaturating amounts of mAb C. These beads precipitate unligated CD13 (by the mAb C bound to the beads), CD13 bound by mAb 452 (because mAb C on the beads will bind to CD13-mAb 452 complexes), and CD13 bound to mAb C (by binding of CD13-mAb C complexes to free protein G sites on the agarose beads). The immunoprecipitates were resolved in SDS-PAGE gels and electrotransferred onto nitrocellulose membranes. The blots were probed with anti-phosphotyrosine antibodies and with anti-CD13 mAb 452. The results showed that both mAb C (Figure 7(b)) and mAb E (not shown) are able to induce tyrosine phosphorylation of CD13 as efficiently as mAb 452. Importantly, when cells were first incubated for 30 min with mAb C followed by stimulation with mAb 452, phosphorylation of CD13 was slightly higher than that induced by mAb 452 alone (Figure 7(b), last lane).

These results show that crosslinking of CD13 by any the anti-CD13 mAbs 452, C, and E is able to stimulate signal transduction events of similar magnitude, suggesting that their different effects on HA of U-937 cells are not related to differences in their ability to stimulate CD13-mediated signal transduction.

### 3.6. MAbs C and E Inhibit CD13-Induced Adhesion of Monocytes to Endothelial Cells* In Vitro*

**Figure 8:**
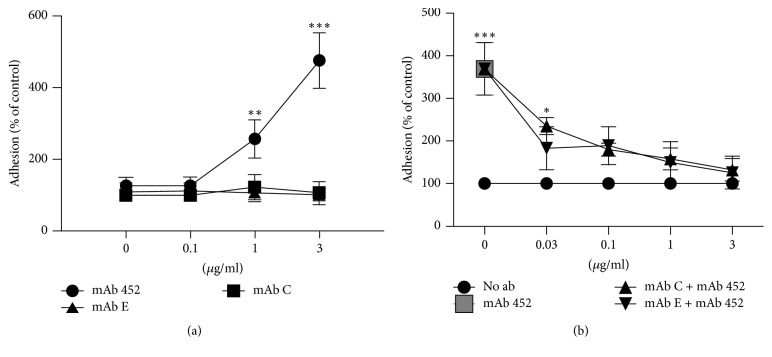
*Effect of anti-CD13 monoclonal antibodies on heterotypic aggregation of human monocytes to HMEC-1 endothelial cells*. (a) CFSE-labeled U-937 cells were incubated with anti-CD13 monoclonal antibodies 452, C, or E for 3 hrs at 37°C. After washing, stimulated monocytes were transferred to monolayers of HMEC-1 cells in 48-well plates and incubated for 15 min at 37°C. Cells were gently washed with warm media and adhered monocytes were quantified with the plate fluorimeter Modulus II. (b) CFSE-labeled monocytes were incubated with different concentrations of anti-CD13 C or E, or with no antibody, for 1 hr at 37°C, and, after washing, mAb 452 (at 3 *µ*g/ml) was added and incubation continued for 3 hours. After washing, stimulated monocytes were transferred to monolayers of HMEC-1 cells in 48-well plates and incubated for 15 min at 37°C. Plates were gently washed with warm media and adhered monocytes were quantified with the plate fluorimeter Modulus II. Data is presented as percentage of antibodies-treated cells attached to HMEC-1 monolayers, compared to the attachment of cells not treated with mAbs. Mean ± SD of three independent experiments. ^*∗*^*p* < 0.05, ^*∗∗*^*p* < 0.01, and ^*∗∗∗*^*p* < 0.0001.

Besides inducing homotypic aggregation of monocytic cells, CD13 crosslinking by mAb 452 has been shown to induce adhesion of monocytes to endothelial cells [19], which is the first step during transendothelial migration of monocytes from blood to inflamed tissues. Thus, we investigated the effect of mAbs C and E on the heterotypic adhesion of U-937 cells to HMEC-1 human endothelial cells. We stimulated human U-937 monocytes with the different anti-CD13 monoclonal antibodies or control IgG and allowed them to adhere to a monolayer of HMEC-1 endothelial cells. Stimulation of U-937 cells with mAb 452 induced a fivefold increase in the attachment of monocytes to endothelial cells, whereas treatment with mAbs C and E did not induce monocyte adhesion (Figure 8(a)). However, when we first incubated the monocytes with the inhibitory mAbs C and E, and then added the mAb 452, we observed a dose-dependent inhibition of the adhesion induced by mAb 452 (Figure 8(b)). Thus, mAbs C and E are able to inhibit the mAb 452-induced heterotypic adhesion of monocytes to endothelial cells.

### 3.7. Anti-CD13 mAbs 452, C, and E Inhibit the Adhesion of Cells Expressing CD13 to Fibronectin

It has been shown that APN/CD13 is the target of tumor homing peptides harboring the NGR sequence motif [14], and that CD13 can bind to fibronectin, which contains four NGR motifs [25]. We were interested in evaluating if the different anti-CD13 mAbs have an effect on CD13-mediated binding of monocytes to fibronectin. However, neither U-937 nor THP-1 monocytic cell lines showed significant binding to fibronectin-coated plastic wells (not shown). Also, CD13 crosslinking by mAb 452 on these cells did not induce binding to fibronectin-coated wells (not shown). This suggests that homotypic and heterotypic cell-cell adhesion induced by mAb 452 is mediated by a mechanism different from that involved in CD13 binding to fibronectin.

**Figure 9:**
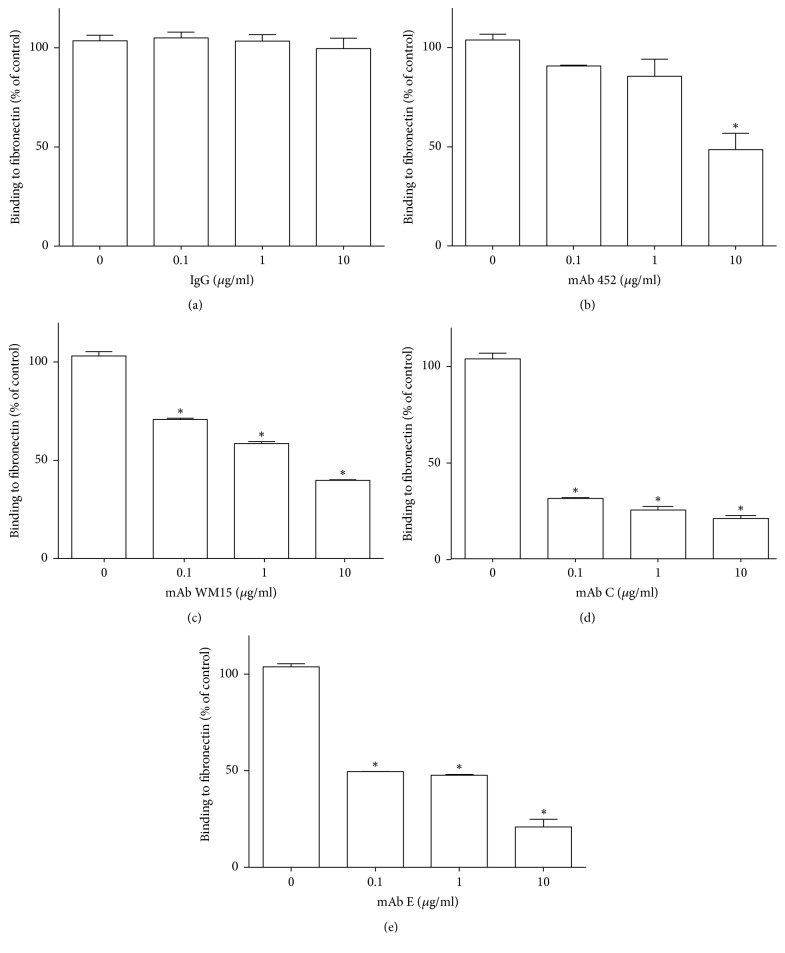
*Monoclonal anti-CD13 antibodies distinctively inhibit binding of HEK-ANPEP cells to fibronectin*. HEK-ANPEP-GFP cells (HEK cells expressing a recombinant CD13 fused to GFP) were incubated with the indicated antibodies (or a control IgG) for 30 minutes at 37°C and transferred to fibronectin-sensitized plates to allow adherence for 15 min at 37°C. Cells were washed very gently and cells attached to the plate were quantitated by measuring the fluorescence in each well with the Cytation 3 plate reader. Data is shown as percentages compared to the number of control cells (without antibody added) attached to similar wells. Average ± SD of three experiments with each condition assayed in triplicate. ^*∗*^*p* ≤ 0.01.

In order to examine the effect of anti-CD13 mAbs on the binding of cells expressing CD13 to fibronectin, we used HEK cells expressing high levels of human CD13 fused to GFP (HEK-ANPEP) on the membrane [18]. Thus, we incubated HEK or HEK-ANPEP cells with the anti-CD13 mAbs 452, WM-15, C, or E, or with a control IgG and, after washing the excess of antibody, transferred the cells to fibronectin-coated plastic wells. While parental HEK cells showed insignificant binding to fibronectin, CD13 expression by HEK-ANPEP cells resulted in a significant increase in the number of cells that adhere to fibronectin-coated wells in our assay conditions (>20,000 cells/well). The different CD13-specific mAbs had different effects on the binding of HEK-ANPEP cells to fibronectin-coated wells (Figure 9). CD13 crosslinking by mAb 452 did not induce an increase in cell binding, but it inhibited cell attachment by about 50% at the highest concentration tested (10 *µ*g/ml). In contrast, mAbs C and E markedly inhibited cell binding at all concentrations tested (0.1, 1.0, and 10.0 *µ*g/ml). MAb WM-15 showed significant inhibition at all concentrations tested, although the degree of inhibition was lower than that induced by mAbs C and E. These results show that although CD13 binding to fibronectin is not induced by mAb 452, mAbs C and E are able to significantly inhibit this interaction.

## 4. Discussion

Aminopeptidase N/CD13 is considered a moonlighting enzyme [3] because in addition to the enzymatic removal of N-terminal amino acids from unsubstituted bioactive peptides, it has an active participation in several functions that are seemingly independent of its enzymatic activity, since they are not affected by chemical inhibitors of the aminopeptidase activity. These later functions include endocytic processes such as cholesterol uptake and entry of certain group 1 coronavirus into cells, as well as participation in cell adhesion and transendothelial migration of monocytes [14, 19, 20]. The ability of CD13 to mediate phagocytosis is also independent of its peptidase activity [18]. The mechanisms by which CD13 participates in processes that are independent of its enzymatic activity are not clearly defined. However, it is known that crosslinking of CD13 by specific monoclonal antibodies initiates signal transduction events, such as a rise in the intracytoplasmic concentration of Ca^2+^ ions, activation of intracellular protein kinases, and phosphorylation of Tyr^6^ in the cytoplasmic portion of CD13 [21, 26]. Thus, it is likely that at least some of these functions depend on CD13 mediating transmembrane signals, as has been shown for CD13-mediated cell adhesion and phagocytosis, which are inhibited by pharmacological inhibitors of tyrosine kinases [17, 18, 21].

We have previously reported that crosslinking of CD13 on the membrane of human monocytic cells by certain monoclonal antibodies (such as mAb 452) induces a signal transduction-dependent change in the adhesion properties of these cells, resulting in homotypic aggregation (HA) of monocytes [17]. Crosslinking of CD13 with the same mAb 452 also induces adhesion of monocytes to HUVEC endothelial cells* in vitro* [19]. Thus, the phenomenon of HA induced by CD13 crosslinking represents a model system in which the molecular mechanisms involved in regulation of cell-cell adhesion by CD13 could be studied. In order to identify membrane molecules involved in cell adhesion processes mediated by CD13, we sought to generate monoclonal antibodies with the ability to modulate the CD13-induced HA of monocytic cells.

We generated two hybridomas that produce the monoclonal antibodies C and E, which bind to monocytic cells and are able to inhibit HA induced by mAb 452. The inhibition was dose-dependent, and mAbs C and E are able to induce the separation of homotypic cell aggregates induced by mAb 452. MAbs C and E recognize a 160 kDa protein that was identified as Aminopeptidase N/CD13 by mass spectrometry. The conclusion that both mAbs bind to CD13 is supported by the observations that both C and E immunoprecipitate the same protein as mAb 452, and their pattern of binding to cells is similar to that of mAb 452: they bind specifically to cells expressing CD13 (U-937, THP-1, HMEC-1, human monocytes from healthy donors) but showed a reduced binding to THP-1 L2 cells (THP-1 cells with reduced expression of CD13). Neither mAb 452 nor mAbs C and E bind to HEK-293 cells, but the three of them bind very efficiently to HEK-293 cells transfected to express human CD13 (HEK-ANPEP cells). Competition experiments showed that the epitope recognized by mAbs C and E on CD13 is different than the one recognized by mAb 452. Interestingly, mAbs C and E seem to bind to the same or to very closely located epitopes, as they inhibit each other's binding, and this could be related to their almost identical biological effects. Likewise, the fact that the epitope recognized by mAb 452 is different from the epitope recognized by mAbs C and E could be related to the distinct effects that they have on CD13 functions.

While mAb 452 has a limited effect on the enzymatic activity of CD13, both mAbs C and E inhibit the aminopeptidase activity of CD13 as efficiently as bestatin, a well-known inhibitor of APN. This inhibition is more easily observed in cells transfected to express high levels of human CD13 on the membrane (Figure 2(d)). Regarding induction of HA of monocytic cells, or induction of heterotypic adhesion of monocytes to endothelial HMEC-1 cells, responses that are efficiently induced by CD13 crosslinking by mAb 452, not only are mAbs C and E unable to induce cell adhesion, but they inhibit homotypic and heterotypic cell adhesion induced by mAb 452 (Figures 1 and 8). Interestingly, despite their different effects on cell adhesion, CD13 crosslinking induced by the three anti-CD13 antibodies 452, C, and E is equally able to induce a fast and significant increase in intracellular Ca^2+^ and tyrosine phosphorylation of CD13, suggesting that although signal transduction is necessary to induce cell adhesion [17, 19], it is clearly not sufficient. The ability of mAb C to initiate signal transduction is in accordance with its previously observed ability to mediate phagocytosis as efficiently as mAb 452 [18].

CD13 is known to interact with the extracellular matrix proteins fibronectin and laminin [25]. Specifically, it was reported that APN/CD13 interacts with a NGR motif in domain V of fibronectin, and binding to extracellular matrix fibronectin through this motif was proposed to be involved in the high migratory competence of tumor cells expressing CD13. Since crosslinking of CD13 by mAb 452 induces monocyte adhesion to CD13-expressing cells very efficiently, we evaluated if it could also promote cell binding to fibronectin. However, in our assay conditions, neither U-937 nor THP-1 monocytic cells showed significant binding to fibronectin-coated wells, and crosslinking of CD13 by mAb 452 was unable to induce adhesion of these cells to fibronectin (not shown). In order to evaluate the effects of the mAbs on cell binding to fibronectin in a system in which this binding was more dependent on CD13, we used HEK-ANPEP cells. In our assays, parental HEK-293 cells showed insignificant binding to fibronectin-coated plates, but transfection of HEK-293 cells for expression of human CD13/APN resulted in a significant increase in binding. In this system, crosslinking of CD13 by mAb 452 did not result in increased cell binding. In contrast, both mAbs C and E, and to a lesser extent mAbs 452 and WM-15, were able to significantly inhibit cell binding to fibronectin. These results suggest that CD13 mediated cell-cell adhesion (which is induced by mAb 452) involves a different mechanism than the one responsible for CD13 binding to fibronectin (which can be inhibited by mAb 452).

Since tumor cell motility has been proposed to involve CD13 binding to NGR motifs on fibronectin [25], and mAbs C and E are able to inhibit CD13-mediated cell binding to fibronectin, one possibility is that mAbs C and E in some way affect the region of CD13 involved in NGR binding, which is the catalytic site [25]. This would be in line with the significant inhibition of CD13 enzymatic activity by mAbs C and E. Moreover, the limited and medium effects of mAb 452 and mAb WM-15, respectively, on cell binding to fibronectin, correlate with their relative efficiency for inhibition of CD13 enzymatic activity. Thus, it could be suggested that binding of mAbs C or E to CD13 either sterically impede the interaction of NGR motifs and substrates with the enzymatic active site, or favor a conformation of the enzyme in which the enzymatic active site is closed.

Although mAbs C and E bind to CD13 at epitopes different from that recognized by mAb 452, CD13 crosslinking by all three mAbs induces signal transduction, as demonstrated by the sharp and significant increase in the intracellular concentration of Ca^2+^ ions and the induction of CD13 phosphorylation induced by the three mAbs. Since mAbs C and E do not induce CD13 internalization, and they do not impede binding of mAb 452, this opens the question of the mechanism by which crosslinking of CD13 by mAb 452 induces HA of monocytes and heterotypic adhesion of monocytes to endothelial cells, whereas crosslinking by mAbs C and E does not induce but, on the contrary, inhibit these adhesion phenomena. We suggest that all our observations could be explained by proposing that the region of CD13 involved in cell adhesion is different from the one involved in binding to NGR motifs. This is consistent with the finding of Ghosh et al. [20], who demonstrated that cell-cell adhesion is mediated by the C terminus of the CD13 molecule (corresponding to 50% of domain VI and domain VII), while the NGR motif binds at the catalytically active site, which is known to be located in a cavity limited by the four extracellular domains (domains IV–VII). We further propose that crosslinking of CD13 by mAb 452 induces a conformation in which the sites on the C terminal half of the molecule that are involved in cell-cell adhesion are exposed and able to mediate interaction with other CD13 molecules on a neighboring cell. Of note, this conformation of CD13 is not the basal one, as cell adhesion is only induced after CD13 crosslinking by certain mAbs (and presumably by a putative CD13 ligand* in vivo*). On the other hand, CD13 crosslinking by mAbs C and E induces dimers with a conformation in which the sites involved in cell-cell adhesion are not exposed. Further support for the hypothesis that the sites involved in cell-cell adhesion are not the same as those involved in binding to fibronectin and NGR motifs comes from the fact that binding of CD13 to fibronectin and NGR motifs is not favored, but rather slightly inhibited, after crosslinking by mAb 452. The conformation imposed by mAbs C and E more strongly inhibits interactions of CD13 with fibronectin, NGR motifs, or substrates, and thus these mAbs are good inhibitors of the enzymatic activity. Distinct conformations of membrane receptors aggregates imposed by different monoclonal antibodies that result in distinct cellular responses have been observed for monoclonal antibodies specific for the Fc*ε*RI on mast cells [27].

The observation that all three mAbs are equally able to induce signal transduction after crosslinking CD13 suggests that aggregation of CD13, and not a specific conformational change, is the critical event necessary to initiate signal transduction. Resolution of the crystallographic structure of CD13/APN [5] revealed that CD13 monomers could exist in either an open or a closed conformation. It was proposed that changes in the distance between the intracellular portions of CD13 when dimers are formed by monomers in an open/open or in a closed/closed conformation (102 versus 54 Å) could be a mechanism used by CD13 to transmit signals across the membrane. Although it is possible that, in aggregates formed by the three mAbs, the intracellular portions of different CD13 molecules are at distances suitable for signal initiation, it is also possible that formation of stable CD13 aggregates is a sufficient signal for initiating transmembrane signaling, as is the case for several receptors of the immune system, in which conformational changes in the extracellular domains of the receptor are not reflected in conformational changes in the cytoplasmic domains, and it is just the aggregation of the intracellular portions which initiates signaling. Resolution of the structure of signaling-competent CD13 aggregates would be necessary to resolve this issue. In summary, although we propose that the conformation of the large extracellular portion of CD13 is different depending on the specific epitope recognized by the different mAbs, aggregation of transmembrane and cytoplasmic segments of CD13 is sufficient to initiate signal transduction.

The moonlighting properties of CD13 might be assumed to involve different parts of its structure. Enzymatic activity and binding to extracellular matrix proteins through NGR motifs is mediated through the catalytic site involving domains IV to VII. Cell-cell adhesion is mediated by the C terminal part (involving half of domain VI and domain VII) while signal transduction depends on the transmembrane and cytoplasmic domains (I and II).

Finally, CD13 is considered a viable target for cancer therapy [10, 28] in view of its participation in processes such as angiogenesis, tumor cell migration, and invasiveness, and the fact that expression of CD13 is dysregulated in several types of cancer cells, contributing in several ways to the malignancy. Several drugs interfering with CD13 functions are being tested for the development of anticancer drugs [1, 29–32]. In this respect, some properties of mAbs C and E, such as the strong inhibition of enzymatic activity and the ability to inhibit cell attachment to extracellular matrix proteins, deserve further study because of their potential to be exploited in cancer therapy.

## Supplementary Materials

Supplementary Materials Figure  1S: binding of mAbs C and E to CD13 does not induce changes in membrane expression of CD13. (A, B) Human U-937 monocytic cells were incubated for 3 h at 4°C (A) or at 37°C (B), with mAbs C and E, a control IgG, or no antibody, at concentrations at which C and E cause total inhibition of HA. Later, cells were transferred to 4°C and fixed. After fixation, CD13 expression was assessed by the binding of mAb 452-FITC, evaluated by flow cytometry. (C, D) U-937 cells were incubated for 3 h at 4°C (C) or at 37°C (D), with mAb 452 (at the optimal concentration for inducing HA), with a control IgG, or with no antibody. Later, cells were transferred to 4°C and fixed. After fixation, CD13 expression was assessed by the binding of mAb C-FITC, which was evaluated by flow cytometry. Histograms of a single representative experiment.
