# Supplementary material for: Distinct Epitopes on CD13 Mediate Opposite Consequences for Cell Adhesion
Source: Biomed Res Int. 2018 Mar 29;2018:4093435. doi: 10.1155/2018/4093435 (PMC5896358; doi:10.1155/2018/4093435)
Supplement: Supplementary Materials — Figure 1S: binding of mAbs C and E to CD13 does not induce changes in membrane expression of CD13. (A, B) Human U-937 monocytic cells were incubated for 3 h at 4°C (A) or at 37°C (B), with mAbs C and E, a control IgG, or no antibody, at concentrations at which C and E cause total inhibition of HA. Later, cells were transferred to 4°C and fixed. After fixation, CD13 expression was assessed by the binding of mAb 452-FITC, evaluated by flow cytometry. (C, D) U-937 cells were incubated for 3 h at 4°C (C) or at 37°C (D), with mAb 452 (at the optimal concentration for inducing HA), with a control IgG, or with no antibody. Later, cells were transferred to 4°C and fixed. After fixation, CD13 expression was assessed by the binding of mAb C-FITC, which was evaluated by flow cytometry. Histograms of a single representative experiment. [file 4093435.f1.docx]

4º C 37º C

B

A

No ab

IgG

mAb C

mAb E

+ mAb452 FITC

452 FITC

D

C

No ab

IgG

mAb 452

+ mAb C FITC

mAb C FITC

**Fig 1S. Binding of mAbs C and E to CD13 do not induce changes in membrane expression of CD13. A,B)** Human U-937 monocytic cells were incubated for 3 h at 4ºC (A) or at 37 ºC (B), with mAbs C, E, a control IgG, or no antibody, at concentrations at which C and E cause total inhibition of HA. Later, cells were transferred to 4º C and fixed. After fixation, CD13 expression was assesed by the binding of mAb 452-FITC, evaluated by flow cytometry. **C,D)** U-937 cells were incubated for 3 h at 4ºC (C) or at 37 ºC (D), with mAb 452 (at the optimal concentration for inducing HA), with a control IgG, or with no antibody. Later, cells were transferred to 4º C and fixed. After fixation, CD13 expression was assesed by the binding of mAb C- FITC, which was evaluated by flow cytometry. Histograms of a single representative experiment.
